# How to Understand “Herd Immunity” in COVID-19 Pandemic

**DOI:** 10.3389/fcell.2020.547314

**Published:** 2020-09-24

**Authors:** Yuanqing Xia, Lumin Zhong, Jingcong Tan, Zhiruo Zhang, Jiajun Lyu, Yiting Chen, Anda Zhao, Lili Huang, Zichong Long, Ning-Ning Liu, Hui Wang, Shenghui Li

**Affiliations:** ^1^School of Public Health, Shanghai Jiao Tong University School of Medicine, Shanghai, China; ^2^International Peace Maternity & Child Health Hospital, Shanghai Jiao Tong University School of Medicine, Shanghai, China; ^3^Department of Epidemiology and Public Health, University College London, London, United Kingdom; ^4^Shanghai Ninth People’s Hospital, Shanghai Jiao Tong University School of Medicine, Shanghai, China; ^5^The Ministry of Education of the People’s Republic of China (MOE)-Shanghai Key Laboratory of Childre’s Environmental Health, Shanghai Jiao Tong University School of Medicine, Shanghai, China

**Keywords:** COVID-19, SARS-CoV-2, herd immunity, outbreak, pandemic

## Abstract

The COVID-19 pandemic has been a global threat. Through rapid and effective surveillance and control, the newly confirmed patients have been fluctuated at a very low level and imported case explained most of them through March, 2020 to the present, indicating China’s response has achieved a stage victory. By contrast, the epidemic of COVID-19 in other countries out of China is bursting. Different countries are adopting varied response strategy in terms of their public health system to prevent the spread. Herd immunity has been a hot topic since the outbreak of COVID-19 pandemic. Can it be a possible strategy to combat COVID-19? To fully interpret the knowledge regarding the term upon the background of COVID-19-related health crisis, we aim to systematically review the definition, describe the effective measures of acquiring herd immunity, and discuss its feasibility in COVID-19 prevention. Findings from this review would promote and strengthen the international cooperation and joint efforts when confronting with COVID-19.

## Introduction

On March 11st, 2020, the world health organization (WHO) declared Coronavirus Disease 2019 (COVID-19) as a global pandemic. By 10am on August 30th, 217 countries or regions had reported confirmed cases, with a total of more than 25,070,000 cases (World Health Organization, 2020b). In early March, British Prime Minister Boris Johnson unveiled UK’s plan to tackle the COVID-19 outbreak through four phases – Contain, Delay, Research and Mitigate. On March 12nd, the Prime Minister announced that the country had switched from the “Contain” to “Delay” phase. On March 13rd, Sir Patrick Vallance, the Government’s chief scientific adviser, mentioned “herd immunity” and pointed out that passively waiting for “herd immunity” would lead to 60% of the population infected with COVID-19. Ever since then, “herd immunity” was top searched through the internet and remains to be a hot issue in debate. Even in April, Sweden’s chief epidemiologist, Dr. Anders Tegnell, said Sweden was tackling the COVID-19 outbreak through “herd immunity.” At approximately the same time, the United States and Australia were also under public scrutiny over whether the two countries were adopting “herd immunity” strategy. The term “herd immunity” has never been noticed by the general public before and this is unprecedented that it comes into our sight. Here, we choose China, the United Kingdom, Sweden, the United States, and Australia as our settings, illustrating the theoretical basis of “herd immunity,” and discuss its feasibility in the fight of curtaining COVID-19 outbreak.

## What Is “Herd Immunity”?

“Herd immunity,” as a concept in immunology, is used to describe the resistance to the spread of a contagious disease within a population or herd. The concept was first proposed in Topley and Wilson (1923) in their publication named the spread of bacterial infection: The problem of “herd immunity.” Herd immunity only exists when a sufficiently high proportion of the population generate immunity against the foreign pathogen so that the probability of transmission between infected and susceptible individuals is reduced (Smith, 2019). In other words, it is becoming difficult for contagious disease to spread between individuals if herd immunity exists as the chain of transmission is broken and the susceptible individuals are protected from infection.

**FIGURE 1:**
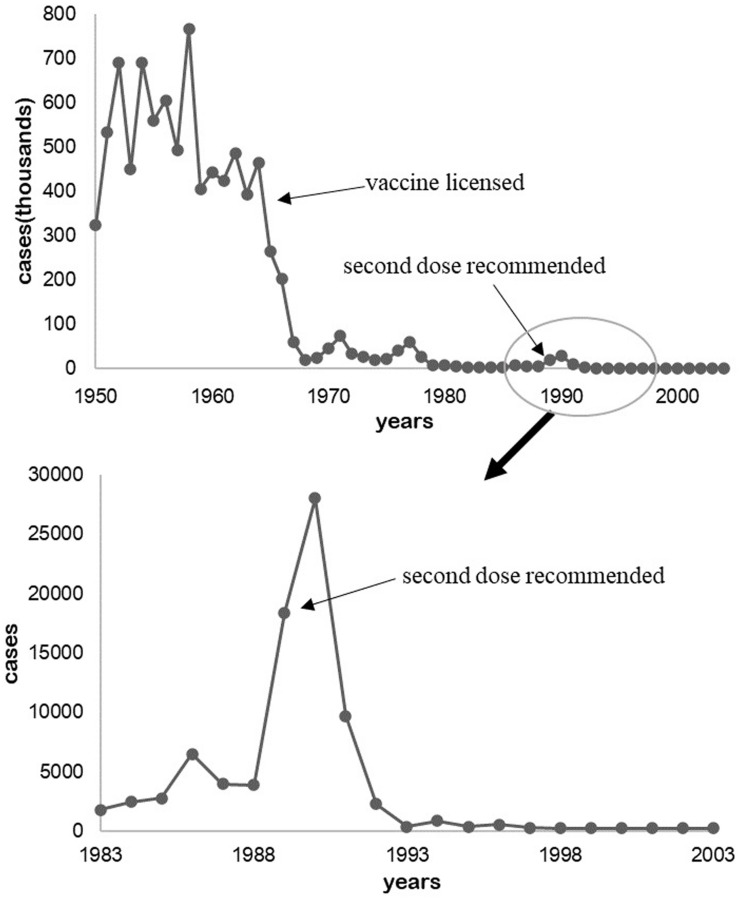
Measles cases in the United States from 1950 to 2004.

In 1933, Dr. Arthur W. Hedrich, health official of Chicago, Illinois, observed the phenomenon that the measles outbreak was prevented after 68% of children were infected between 1900 and 1930 in Boston, Massachusetts (Fine, 1993). The number of cases were kept to a low level after the measles vaccine was legalized in 1964 and the second dose was inoculated till the late 1980s (Figure 1; CDC, 1993; McNabb et al., 2007).

## How to Achieve Herd Immunity?

Herd immunity is based on individual immunity which refers to a physiological function that the body’s immune system recognizes and differentiates its own and alien substances and eliminates the antigenic substances (such as bacteria and viruses) through immune response to maintain health. It may be built up by confronting a disease or infection in the past and recovering from it. Immunity can also be induced by vaccination. Herd immunity is usually achieved by vaccination (e.g., smallpox vaccine) or by lots of people being infected with the contagious disease (e.g., influenza).

## COVID-19 and Herd Immunity

### The History of Herd Immunity

There are many examples in human history of blocking or even eliminating infectious diseases through herd immunity (Fine et al., 2011). Smallpox is considered to be among the most deadly infectious diseases human are generally prone to be infected. Its spread in populations initiated for thousands of years from ancient times to the recent human history (Theves et al., 2016). In 1979, smallpox was officially declared eradicated based on herd immunity achieved by intensive vaccination campaigns (Lane, 2006). Similarly, rinderpest, a highly contagious disease, was eradicated in 2011 through herd immunity in animals (Tounkara and Nwankpa, 2017). Other ubiquitous diseases such as measles, rubella, pertussis are not eradicated yet, herd immunity is maintained by keeping the proportion immune above some threshold to protect susceptible individuals (Black, 1982; Assaad, 1983; Fine et al., 2011). Up to now the number of cases were kept in a low level (Adams et al., 2017). In 1988, the incidence of measles in the United States fell to 1.3 cases per million following the introduction of measles vaccines by initiating two measles elimination efforts, and reemergence of indigenous transmission in the United States finally disappeared since 2000 (Katz and Hinman, 2004; Phadke et al., 2016). After the whole-cell pertussis vaccines were widely used into routine childhood immunization in the mid- 1940s, there was a markable reduction in the pertussis incidence, from 150,000 to 260,000 cases to a nadir of just 1010 cases in 1976 annually (CDC, 1922-2018; Phadke et al., 2016).

### Possible Outcomes After Herd Immunity

Since there’s no approved vaccine for COVID-19 yet, the herd immunity cannot be achieved by vaccination. If herd immunity is derived from natural infection, what is the proportion of a population that need to be immunized in order to achieve the effect of protection? We can estimate this ratio based on the basic infection number (R_0_, the expected average number of additional cases that one case will generate over the course of its infectious period in an otherwise uninfected and generally susceptible population) of COVID-19. The R_0_s of some common vaccine-preventable contagious diseases are shown in Table 1 (Fine et al., 2018). Based on the formula of herd immunity threshold (threshold = 1–1/R_0_) (Fine et al., 2011), and the R_0_ of COVID-19 being 2.27 (Zhang et al., 2020), only when about 56% of population get specific immunity to SARS-CoV-2, then transmission-blocking can be achieved with herd immunity. Herd immunity in measles suggests the whole population is protected from emerging infections when 90% or more are immunized, whether by vaccination or recovery from natural infection. However, in the case of COVID-19, there are two outcomes when people get naturally infected – recovery and death. It is unclear whether those who are cured are free of the virus and exempted from the contagious, which indicates that the percentage of infected people would be more than 60–70%.

**TABLE 1:** R_0_ and threshold value of herd immunity of common vaccine-preventable contagious diseases.

Disease	Route of transmission	R_0_	Herd immunity threshold
Diphtheria	Saliva	6–7	83–85%
Measles	Air borne	12–18	92–94%
Mumps	Droplet spread	4–7	75–86%
Pertussis	Droplet spread	12–17	80–94%
Poliomyelitis	Fecal-oral transmission	5–7	50–95%
Rubella	Droplet spread	5–7	83–85%
Smallpox	Contact transmission	6–7	80–85%

According to an epidemiological analysis of COVID-19 in China, COVID-19 can cause about 15% severe cases and a 2% death rate (World Health Organization., 2020a; Zhang et al., 2020). Particularly, the projections above were based on the existing data in China where the overall isolation and the centralized allocation of medical resources of the whole country are adopted. Without effective medical resources and isolation interventions, natural infection may result in a more severe mortality rate.

What will be the cost of the government’s “herd immunity” or “mitigate” strategy? A simulation study about the pandemic trend by epidemiological model found that an estimated number of 510,000 British people will die if nothing is carried out, and about 250,000 British people would also die if mitigation measures were maximized (Ferguson et al., 2020). The study predicted that the peak in mortality would occur after 3 months and, given the estimated R_0_ of 2.4, 81% of United Kingdom and United States populations would be infected.

### Herd Immunity Lessons From Other Viruses

The length of duration of herd immunity was challenged by immune senescence, and the breadth of duration was challenged by antigenic diversity of a pathogen (Mallory et al., 2018). Over time, the progressive loss of responsiveness to a pathogen and the decreased antibody titer or cellular responses would result in loss of immunity. In the early 21th century, measles infections peaked in Chinese middle-aged adults after re-introduction of the wild-type virus, who had been immunized by early-age vaccination and then boosted with attenuated virus after a time interval (He et al., 2013). And this phenomenon also occurred in South Korea (Kang et al., 2017).

Generally, a viral species especially RNA viruses, consists of multiple antigenically distinct variants resulted from antigenic drift, antigenic shift, and recombination (Pica and Palese, 2013; Payne, 2017). However, most RNA polymerases lack a proof-reading function to solve it. It poses challenging obstacles in eliciting broad immunity through vaccination with a single serotype of attenuated virus, as evidenced by Norovirus (Debbink et al., 2014), dengue (Midgley et al., 2011), and influenza (Wu et al., 2017). Moreover, vaccination with a single serotype may increase the severity of a secondary infection, which ever occurred in Dengue virus with four serotypes (de Alwis et al., 2014). Similarly, vaccination with the bivalent HPV vaccine caused decline in the prevalence of HPV types 16 and 18 and cross-protection against non-vaccine types HPV 31, 33, and 45, but increased prevalence of non-vaccine, non–cross-protective HPV types (Brisson et al., 2016; Cameron et al., 2016; Ribeiro et al., 2020).

### Being a Threat to the World

We are now living in an era of “the global village” with constant flux of large populations. There will be an “Immunity gap” if the majority of people gain antiviral immunity to SARS-CoV-2 in particular countries by natural infection but not in the other countries. Once the solid growth of economy is restored and traffic controls are lifted, there might be large-scale international transmissions with unsatisfactory outcomes. For example, in the early 16th century, the smallpox virus ever killed 3 million Indians who had never been exposed to it after it was introduced to America by European colonists who had been immunized against the smallpox virus (Eyler, 2003).

Besides, taking measles as another example, measles epidemics continues to occur even when the measles vaccine is widespread (Smiianov et al., 2019). In 2008, an unvaccinated 7-year-old boy contracted measles and infected 11 children, after returning home from a family vacation in Switzerland to San Diego, a city with a 95% measles vaccination rate (Sugerman et al., 2010). That proportion, by the concept of herd immunity, should be enough to keep measles at bay and protect those unvaccinated. This outbreak is mainly due to failure to vaccinate and importation of cases (Haralambieva et al., 2019). In fact, although the average vaccination rate may be high across the county, it varied locally. Rates in some neighborhoods may be far below the necessary threshold to achieve herd immunity (Peeples, 2019). Various studies have estimated that 2–10% of individuals vaccinated against measles may not develop immunity, allowing a gradual accumulation of susceptible individuals to infection and subsequently outbreaks (Poland and Jacobson, 1994; HaralambievaI, Ovsyannikova et al., 2011; Whitaker and Poland, 2014; Haralambieva et al., 2015). Since the mobility of individuals with measles across global, it is hard to avoid imported infections.

The epidemics above can be addressed by vaccinating and treating patients. But now there’s no approved vaccine for COVID-19, many people will inevitably die once the emergence of an epidemic occurs. The total number of imported cases of COVID-19 in China reached 2482 as that of August 30th, which poses a threat to the country.

### Potential Risk of Virus Mutation

To survive and escape the herd immunity, the virus may fight by gene mutation and then the original immune system won’t recognize the mutated virus and the herd immunity will thus be ineffective. In this case the viruses can be divided into two categories – DNA and RNA viruses. DNA viruses are stable with low possibility of mutation while RNA viruses are unstable and prone to mutate (Gelderblom, 1996). The SARS-CoV-2 is an RNA virus with a high potential risk of mutation (Phan, 2020). Homologous recombination may result in the cross-species transmission of SARS-CoV-2 (Ji et al., 2020). Population genetic analysis of 103 SARS-CoV-2 genomes showed that SARS-CoV-2 could be categorized into two major types: L and S. The S type was relatively an ancestral version while the L type was found to be more prevalent than S type in the early stage of the outbreak in Wuhan. Besides, 149 sites across 103 sequenced strains were identified (Tang et al., 2020). Recently, a phylogenetic analysis based on 377 complete genomic sequences of the SARS-COV-2 suggested that the virus was actively evolving in human hosts from December 2019 to March 2020 (Li et al., 2020). The highly frequent mutations resulted in at least 5 differentiated SARS-CoV-2 strains and are predicted to enhance the virulence and transmission (Singh et al., 2020; Wang et al., 2020). However, this is a relatively small number of mutations passed through over 300,000 people. At this point, it is still believed that the mutation rate remains low. The analysis from existing study showed that the polyprotein 1ab(pp1ab), the largest protein of coronaviruses, hadn’t changed in most isolates during the outbreak (Cárdenas-Conejo et al., 2020). In addition, the critical mechanism for SARS-CoV-2 infection is through S protein binding to the human ACE2 (Lu et al., 2020), and there is no evidence that the binding site of S protein was mutated. Thus the SARS-CoV-2 should be relatively genomically stable.

### Epidemiology of COVID-19 in the United Kingdom, Sweden, the United States, and Australia

#### United Kingdom

The first two cases were confirmed in the United Kingdom on 31 January, 2020. One of them is an international student at York University and the other is the relative of the student. On March 6th, the number of covid-19 cases in the United Kingdom showed a rapid increase, and the number of new cases in a single day broke new record, reaching 36, with a total of 87 confirmed cases. United Kingdom was the first country that mentioned “herd immunity,” but in fact United Kingdom did not adopt “herd immunity” as their strategy against the virus. At present, the total number of confirmed cases in the United Kingdom got stabilized and the outbreak has shown a controllable trend. As of August 30st, 2020, the total number of COVID-19 infections in the United Kingdom reached 334,915. The total number of deaths is 41585.

#### Sweden

The epidemic in Sweden has come under an intense scrutiny since April, when the country’s chief epidemiologist, Dr. Anders Tegnell, announced the country was adopting “herd immunity” to fight against the COVID-19 outbreak. In Sweden, from 31 January to 30 August 2020, there have been 87,072 confirmed cases of COVID-19, along with 5,821 deaths. The number of newly infected cases has become decreased in June but surged again in July. Sweden’s death toll is more than three times than that of it’s neighbors, Denmark, Finland, and Norway, combined, where lockdown was adopted.

#### US

Due to the negative response to fight against COVID-19, the epidemic situation in United States had attracted global attention and more and more people began to doubt whether they are adopting “herd immunity” as response strategy or not. Based on the monitoring data of COVID-19 from WHO, we observed that the cases newly infected in the United States has been decreased in June but went up in July, which should be related to the economy reactivation. Currently, the United States has the highest number of confirmed cases in the world, with the huge number of new cases every day.

#### Australia

Similarly, Australia was also exposed to public scrutiny whether they had adopted “herd immunity”. From the WHO data we can see that the cases newly infected in Australia went up again after complete suppression.

**FIGURE 2:**
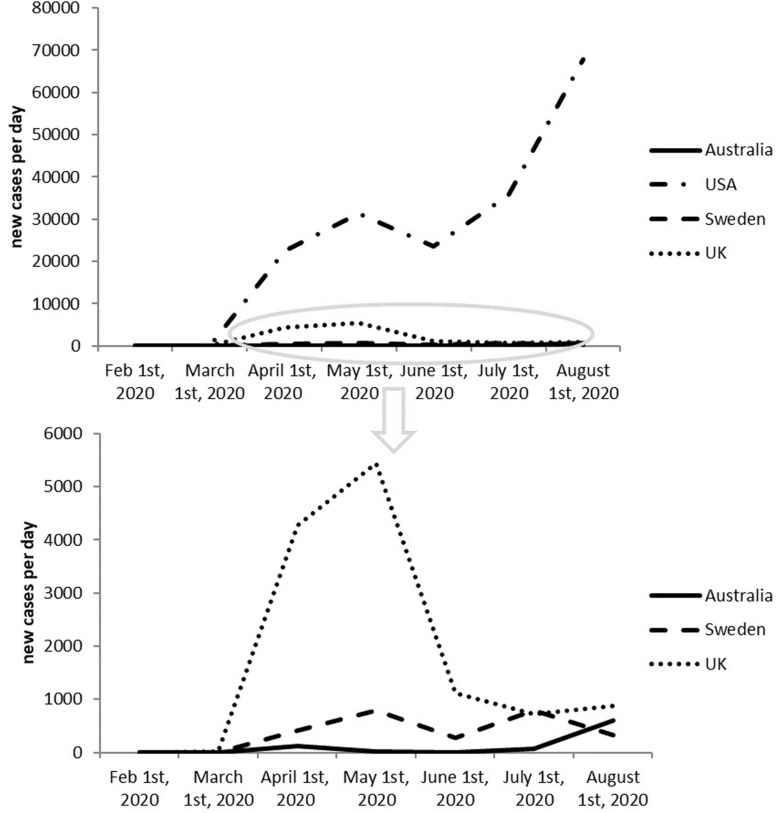
Newly reported cases in last 6 months of United Kingdom, Sweden, United States, and Australia.

It seems like a risky move to adopt herd immunity as a national strategy. And honestly, Figure 2 shows that herd immunity doesn’t seem to be working. In contrast, United Kingdom, has been witnessing newly infected cases decreasing since May as a result of adopting positive measures against COVID-19.

## Discussion

Herd immunity can be safely achieved only if it is actively obtained through vaccination. It is not desirable or feasible to achieve herd immunity through natural infection of the population.

To combat with the pandemic of COVID-19, the Chinese government put people’s life and health above everything else by taking the most proactive and decisive measures and provided valuable successful experiences for the global “anti-pandemic” battle, such as differentiated isolation strategies and clinical treatments according to different symptoms. In fact, United Kingdom did not adopt “herd immunity” as their strategy against the virus.

We are pious toward our history in order to stay alert and take effective action if needed. COVID-19, SARS and MERS-CoV all belong to human coronaviruses but COVID-19 has caused more deaths than combination of the other two despite the fact that COVID-19 has a lower fatality rate (Mahase, 2020). The history of combating with SARS as well as MERS-CoV has provided much valuable information on the COVID-19 pandemic control. Epidemiological research helps unveil some traits of the viruses but yet there are so many questions remain unanswered.

**FIGURE 3:**
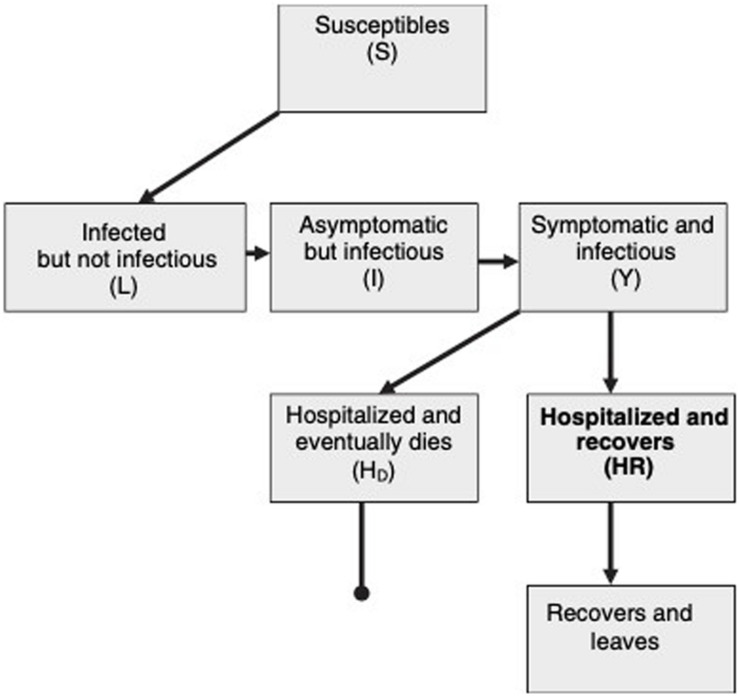
Schematic flow diagram of SARS transmission within a population (Riley et al., 2003).

Figure 3 shows the schematic flow diagram of virus transmission within a population. Susceptible individuals, first become infected and then enter into a latent class, L. They can then progress to a short asymptomatic and potentially infectious stage, I, before the onset of symptoms and the progression to class Y. This diagram assumes that every infected individual eventually goes to hospital and either recovers(HR) or dies(HD) (Riley et al., 2003).

A review on MERS-CoV raised a set of unanswered questions concerning an emerging virus outbreak, such as: the exact routes of the transmission and the incubation period. We don’t know yet whether the virus can cause mild and unrecognized diseases and it is also possible that what is reported only represents the tip of an iceberg. The same questions also exist with COVID-19 (Al-Tawfiq, 2013).

However, the strict isolation strategy adopted by China was proved to be effective in the fight against the COVID-19 outbreak. Although the epidemic is currently recurring in some cities at present, all of them are within controllable range. The result of South Korea’s fight against the epidemic also confirmed the effectiveness of the isolation strategy.

In contrast, India has not implemented strict isolation interventions. Since May, the number of new cases per day has continued to increase, even reaching to 70,000. Currently, the total number of cases reached to 3.4 million, ranking third in the world. The same situation also occurs in Brazil and Mexico, which rank second and eighth globally in total cases.

Iran and Iraq had already controlled the epidemic through strict isolation strategy in the early stage. Recently, due to deregulation and imported cases, the epidemic rebounded again and started to get a little out of control.

Taken together, before the emergence of vaccines, isolation is the best and most effective way to fight against the pandemic. The COVID-19 pandemic has been a global public health event, and no country can survive alone. We need to prepare for long-term battles, emphasizing adopting strict isolation measures in severe countries, and resuming work and production without deregulation in countries where the epidemic has improved.

## Author Contributions

SL, HW, and N-NL contributed to conception and design of the study. YX and LZ wrote the first draft of the manuscript. JT wrote sections of the manuscript. ZZ, JL, YC, AZ, LH, and ZL contributed to the acquisition of data for the work. All authors contributed to manuscript revision, read and approved the submitted version.

## Conflict of Interest

The authors declare that the research was conducted in the absence of any commercial or financial relationships that could be construed as a potential conflict of interest.

## References

[B1] Adams D. A. Thomas K. R. Jajosky R. A. Foster L. Baroi G. Sharp P. (2017). Summary of notifiable infectious diseases and conditions - United States, 2015. *MMWR* 64 1–143. 10.15585/mmwr.mm6453a1 28796757

[B2] Al-Tawfiq J. A. (2013). Middle east respiratory syndrome-coronavirus infection: an overview. *J. infect. Public Health* 6 319–322. 10.1016/j.jiph.2013.06.001 23999347 PMC7102700

[B3] Assaad F. (1983). Measles: summary of worldwide impact. *Rev. Infect. Dis.* 5 452–459. 10.1093/clinids/5.3.452 6878998

[B4] Black F. L. (1982). The role of herd immunity in control of measles. *Yale J. Biol. Med.* 55 351–360. 7180027 PMC2596463

[B5] Brisson M. Benard E. Drolet M. Bogaards J. A. Baussano I. Vanska S. (2016). Population-level impact, herd immunity, and elimination after human papillomavirus vaccination: a systematic review and meta-analysis of predictions from transmission-dynamic models. *Lancet. Public Health* 1 e8–e17. 29253379 10.1016/S2468-2667(16)30001-9 PMC6727207

[B6] Cameron R. L. Kavanagh K. Pan J. Love J. Cuschieri K. Robertson C. (2016). Human papillomavirus prevalence and herd immunity after introduction of vaccination program, Scotland, 2009-2013. *Emerg. Infect. Dis.* 22 56–64. 10.3201/eid2201.150736 26692336 PMC4696690

[B7] Cárdenas-Conejo Y. Liñan-Rico A. García-Rodríguez D. A. Centeno-Leija S. Serrano-Posada H. (2020). An exclusive 42 amino acid signature in pp1ab protein provides insights into the evolutive history of the 2019 novel human-pathogenic coronavirus (SARS-CoV-2). *Jo. Med. Virol.* 92 688–692. 10.1002/jmv.25758 PMC7228214 32167166

[B8] CDC (1993). Measles–United States, 1992. *MMWR* 42 378–381. 8487747

[B9] CDC (1922-2018). *National Notifiable Diseases Surveillance System. Surveillance and Reporting: Pertussis Cases by Year, passive reports to Public Health Service.* Atlanta, GA: CDC.

[B10] de Alwis R. Williams K. L. Schmid M. A. Lai C. Y. Patel B. Smith S. A. (2014). Dengue viruses are enhanced by distinct populations of serotype cross-reactive antibodies in human immune sera. *PLoS pathogens* 10:e1004386. 10.1371/journal.ppat.1004386 25275316 PMC4183589

[B11] Debbink K. Lindesmith L. C. Ferris M. T. Swanstrom J. Beltramello M. Corti D. (2014). Within-host evolution results in antigenically distinct GII.4 noroviruses. *J. Virol.* 88 7244–7255. 10.1128/jvi.00203-14 24648459 PMC4054459

[B12] Eyler J. M. (2003). Smallpox in history: the birth, death, and impact of a dread disease. *J. Lab. Clin. Med.* 142 216–220. 10.1016/s0022-2143(03)00102-1 14625526

[B13] Ferguson N. M. Laydon D. Nedjati-Gilani G. Imai N. Ainslie K. Baguelin M. (2020). *Impact of Non-Pharmaceutical Interventions (NPIs) to Reduce COVID-19 Mortality and Healthcare Demand.* Available online at: https://www.imperial.ac.uk/media/imperial-college/medicine/sph/ide/gida-fellowships/Imperial-College-COVID19-NPI-modelling-16-03-2020.pdf (Accessed March 16, 2020) 10.1007/s11538-020-00726-x PMC7140590 32270376

[B14] Fine P. Eames K. Heymann D. L. (2011). “Herd immunity”: a rough guide. *Clin. Infect. Dis.* 52 911–916. 10.1093/cid/cir007 21427399

[B15] Fine P. E. (1993). Herd immunity: history, theory, practice. *Epidemiol. Rev.* 15 265–302. 10.1093/oxfordjournals.epirev.a036121 8174658

[B16] Fine P. E. M. Mulholland K. Scott J. A. Edmunds W. J. (2018). “Community protection,” in *Vaccines*, 7th Edn, eds Plotkin S. A. Orenstein W. A. Offit P. A. Edwards K. M. (Amsterdam: Elsevier), 1512–1531.

[B17] Gelderblom H. R. (1996). “Structure and classification of viruses,” in *Medical Microbiology*, ed. Baron S. (Galveston, TX: University of Texas Medical Branch at Galveston). 21413309

[B18] Haralambieva I. H. I Ovsyannikova G. O’Byrne M. Pankratz V. S. Jacobson R. M. Poland G. A. (2011). A large observational study to concurrently assess persistence of measles specific B-cell and T-cell immunity in individuals following two doses of MMR vaccine. *Vaccine* 29 4485–4491. 10.1016/j.vaccine.2011.04.037 21539880 PMC3117252

[B19] Haralambieva I. H. Kennedy R. B. I Ovsyannikova G. Schaid D. J. Poland G. A. (2019). Current perspectives in assessing humoral immunity after measles vaccination. *Expert Rev. vaccines* 18 75–87. 10.1080/14760584.2019.1559063 30585753 PMC6413513

[B20] Haralambieva I. H. Kennedy R. B. I Ovsyannikova G. Whitaker J. A. Poland G. A. (2015). Variability in humoral immunity to measles vaccine: new developments. *Trends Mol. Med.* 21 789–801. 10.1016/j.molmed.2015.10.005 26602762 PMC4679650

[B21] He H. Chen E. F. Li Q. Wang Z. Yan R. Fu J. (2013). Waning immunity to measles in young adults and booster effects of revaccination in secondary school students. *Vaccine* 31 533–537. 10.1016/j.vaccine.2012.11.014 23159458

[B22] Ji W. Wang W. Zhao X. Zai J. Li X. (2020). Cross-species transmission of the newly identified coronavirus 2019-nCoV. *J. Med. Virol.* 92 433–440. 10.1002/jmv.25682 31967321 PMC7138088

[B23] Kang H. J. Han Y. W. Kim S. J. Kim Y. J. Kim A. R. Kim J. A. (2017). An increasing, potentially measles-susceptible population over time after vaccination in Korea. *Vaccine* 35 4126–4132. 10.1016/j.vaccine.2017.06.058 28669617

[B24] Katz S. L. Hinman A. R. (2004). Summary and conclusions: measles elimination meeting, 16-17 March 2000. *J. Infect. Dis.* 189(Suppl. 1), S43–S47. 15106088 10.1086/377696

[B25] Lane J. M. (2006). Mass vaccination and surveillance/containment in the eradication of smallpox. *Curr. Top. Microbiol. Immunol.* 304 17–29. 10.1007/3-540-36583-4_2 16989262 PMC7120753

[B26] Li Y. Liu B. Cui J. Wang Z. Shen Y. Xu Y. (2020). Similarities and evolutionary relationships of COVID-19 and related viruses. *arXiv [Preprint].* Available online at: https://arxiv.org/abs/2003.05580 (accessed March 26, 2020).

[B27] Lu R. Zhao X. Li J. Niu P. Yang B. Wu H. (2020). Genomic characterisation and epidemiology of 2019 novel coronavirus: implications for virus origins and receptor binding. *Lancet* 395 565–574. 10.1016/s0140-6736(20)30251-8 32007145 PMC7159086

[B28] Mahase E. (2020). Coronavirus covid-19 has killed more people than SARS and MERS combined, despite lower case fatality rate. *BMJ* 368:m641. 10.1136/bmj.m641 32071063

[B29] Mallory M. L. Lindesmith L. C. Baric R. S. (2018). Vaccination-induced herd immunity: successes and challenges. *J. allergy Clin. Immunol.* 142 64–66. 10.1016/j.jaci.2018.05.007 29803799 PMC6433118

[B30] McNabb S. J. Jajosky R. A. Hall-Baker P. A. Adams D. A. Sharp P. Anderson W. J. (2007). Summary of notifiable diseases — United States, 2005. *MMWR* 54 1–92. 17392681

[B31] Midgley C. M. Bajwa-Joseph M. Vasanawathana S. Limpitikul W. Wills B. Flanagan A. (2011). An in-depth analysis of original antigenic sin in dengue virus infection. *J. Virol.* 85 410–421. 10.1128/jvi.01826-10 20980526 PMC3014204

[B32] Payne S. (2017). *Viruses: From Understanding to Investigation.* Cambridge: Academic Press.

[B33] Peeples L. (2019). Rethinking herd immunity. *Nature Med.* 25 1178–1180. 10.1038/s41591-019-0515-2 31227810

[B34] Phadke V. K. Bednarczyk R. A. Salmon D. A. Omer S. B. (2016). Association between vaccine refusal and vaccine-preventable diseases in the united states: a review of measles and pertussis. *JAMA* 315 1149–1158. 10.1001/jama.2016.1353 26978210 PMC5007135

[B35] Phan T. (2020). Genetic diversity and evolution of SARS-CoV-2. *Infect. Genet. Evol.* 81:104260. 10.1016/j.meegid.2020.104260 32092483 PMC7106203

[B36] Pica N. Palese P. (2013). Toward a universal influenza virus vaccine: prospects and challenges. *Annu. Rev. Med.* 64 189–202. 10.1146/annurev-med-120611-145115 23327522

[B37] Poland G. A. Jacobson R. M. (1994). Failure to reach the goal of measles elimination. Apparent paradox of measles infections in immunized persons. *Arch. Intern. Med.* 154 1815–1820. 10.1001/archinte.1994.00420160048006 8053748

[B38] Ribeiro G. S. Hamer G. L. Diallo M. Kitron U. Ko A. I. Weaver S. C. (2020). Influence of herd immunity in the cyclical nature of arboviruses. *Curr. Opin. Virol.* 40 1–10. 10.1016/j.coviro.2020.02.004 32193135 PMC7434662

[B39] Riley S. Fraser C. Donnelly C. A. Ghani A. C. Abu-Raddad L. J. Hedley A. J. (2003). Transmission dynamics of the etiological agent of SARS in Hong Kong: impact of public health interventions. *Science* 300 1961–1966. 10.1126/science.1086478 12766206

[B40] Singh H. Singh J. Khubaib M. Jamal S. Sheikh J. A. Kohli S. (2020). Mapping the genomic landscape & diversity of COVID-19 based on >3950 clinical isolates of SARS-CoV-2: likely origin & transmission dynamics of isolates sequenced in India. *Indian J. Med. Res.* 151 474–478. 32474554 10.4103/ijmr.IJMR_1253_20 PMC7530457

[B41] Smiianov V. A. Kurhanska V. A. Smiianova O. I. (2019). Measles outbreaks: they are preventable but keep progressing dangerously. *Wiadomosci lekarskie* 72 2145–2148. 31860862

[B42] Smith D. R. (2019). Herd Immunity. The veterinary clinics of North America. *Food Anim. Pract.* 35 593–604. 10.1016/j.cvfa.2019.07.001 31590904

[B43] Sugerman D. E. Barskey A. E. Delea M. G. I Ortega-Sanchez R. Bi D. Ralston K. J. (2010). Measles outbreak in a highly vaccinated population, San Diego, 2008: role of the intentionally undervaccinated. *Pediatrics* 125 747–755. 10.1542/peds.2009-1653 20308208

[B44] Tang X. Wu C. Li X. Song Y. Yao X. Wu X. (2020). On the origin and continuing evolution of SARS-CoV-2. *Natl. Sci. Rev.* 3: nwaa036. 10.1093/nsr/nwaa036 PMC7107875 34676127

[B45] Theves C. Crubezy E. Biagini P. (2016). History of smallpox and its spread in human populations. *Microbiol. Spectr.* 4:1. 10.1128/microbiolspec.PoH-0004-2014 27726788

[B46] Topley W. W. Wilson G. S. (1923). The spread of bacterial infection. the problem of herd-immunity. *J. Hyg.* 21 243–249. 10.1017/s0022172400031478 20474777 PMC2167341

[B47] Tounkara K. Nwankpa N. (2017). Rinderpest experience. *Rev. Sci. Tech.* 36 569–578. 30152462 10.20506/rst.36.2.2675

[B48] Wang M. Li M. Ren R. Li L. Chen E. Q. Li W. (2020). International expansion of a novel SARS-CoV-2 mutant. *J. Virol.* 94 e567–e520. 10.1128/JVI.00567-20 PMC7307084 32269121

[B49] Whitaker J. A. Poland G. A. (2014). Measles and mumps outbreaks in the United States: think globally, vaccinate locally. *Vaccine* 32 4703–4704. 10.1016/j.vaccine.2014.06.088 24992719

[B50] World Health Organization. (2020a). *Report of the WHO-China Joint Mission on Coronavirus Disease 2019 (COVID-19).* Geneva: World Health Organization.

[B51] World Health Organization (2020b). *WHO Coronavirus Disease (COVID-19) Dashboard.* Geneva: World Health Organization.

[B52] Wu N. C. Zost S. J. Thompson A. J. Oyen D. Nycholat C. M. McBride R. (2017). A structural explanation for the low effectiveness of the seasonal influenza H3N2 vaccine. *PLoS Pathogens* 13:e1006682. 10.1371/journal.ppat.1006682 29059230 PMC5667890

[B53] Zhang S. Diao M. Yu W. Pei L. Lin Z. Chen D. (2020). Estimation of the reproductive number of novel coronavirus (COVID-19) and the probable outbreak size on the diamond princess cruise ship: a data-driven analysis. *Int. J. Infect. Dis.* 93 201–204. 10.1016/j.ijid.2020.02.033 32097725 PMC7110591

